# The Nutritional Issue of Older People Receiving Home-Delivered Meals: A Systematic Review

**DOI:** 10.3389/fnut.2021.629580

**Published:** 2021-03-04

**Authors:** Ségolène Fleury, Paul Tronchon, Juliane Rota, Charlotte Meunier, Oliver Mardiros, Virginie Van Wymelbeke-Delannoy, Claire Sulmont-Rossé

**Affiliations:** ^1^Centre des Sciences du Goût et de l'Alimentation, AgroSup Dijon, Centre National de la Recherche Scientifique, Institut National de Recherche pour l'Agriculture, l'Alimentation et l'Environnement, Université de Bourgogne Franche-Comté, Dijon, France; ^2^Saveurs et Vie, Orly, France; ^3^Centre Hospitalier Universitaire Dijon Bourgogne, Unité de Recherche Pôle Personnes Âgées, Dijon, France

**Keywords:** aged, older adults, meals-on-wheels, malnutrition, food intake, body weight

## Abstract

**Background:** Setting up a home-delivered meal service often allows older people suffering from physical and/or cognitive disabilities to stay at home. However, older people who delegate their food activities (food purchasing, cooking…) have been reported to have a worse nutritional status than people who take care of their food activities. In this context, we will conduct a systematic review of all studies related to the nutritional issue in home-delivered meal older recipients.

**Methods:** In June 2020, we searched 3 databases (Pubmed, Web of Science, EMBASE) to identify studies from all years on older adults at home and receiving home-delivered meal services (*population*). The following *outcomes* were considered: nutritional status (Body Mass Index, weight, undernutrition) and nutritional intake. Any nutritional *intervention, comparator*, and *study design* were relevant for inclusion.

**Results:** Forty-eight original studies met the inclusion criteria, most of them being published after the year 2000 (*n* = 34) and undertaken in the USA (*n* = 32). The selection includes 30 cross-sectional and 18 longitudinal studies. The main findings of this review are the following: (1) home-delivery meal older recipients are at high risk of undernutrition; (2) providing home-delivery meals may improve the nutritional status and nutrient intake; (3) this improvement is even higher when the home-delivery meal service is improved, for instance by providing dietetic counseling or adding supplementary snacks/meals or enriched food. However, even an improved service does not allow all the older recipients meeting their recommended nutritional allowance.

**Conclusion:** This review reveals a need to further develop strategies allowing home-delivery meal older recipients to fulfill their nutritional needs. From a methodological point of view, there is a need to describe in more detail the home-delivered services provided to studies' participants to better consider meal frequency and meal content in the results.

## Introduction

Home-delivered meal (HDM) services for older adults and/or disabled people were introduced in the United Kingdom during the Second World War, providing meals for people who could no longer prepare food for themselves. Subsequently, this type of service spread first to the United States, Ireland, Australia, and then more generally to the other industrialized countries (1, 2). In the Netherlands, home-delivered meal services seem to be mainly funded by private corporations and not by health insurance or social funds. Conversely, in the United States, home-delivered meal services mainly concern elderly people with financial difficulties and is funded under the Older Americans Act (OAA) (3). Finally, in emerging countries such as Korea and Hong Kong, it is most common to make delivery of fresh products to the elderly (4). In the future, demands to this services that enable seniors to remain residing in their homes can be expected to increase. Indeed, numerous countries in the world are experiencing a tremendous increase of the older population, and notably an increase of the “very old” population, namely people aged 80 or over. The number of 80 and over is expected to increase from 126 million in 2015 to 202 million in 2030 and 426 million in 2050 (5). These “very old” people are also the ones who present the poorest health and accumulate the severest disabilities. Consequently, they are more likely to ask support from care services. More recently, according to home-delivered meal companies, the lockdown due to the Covid-19 epidemic led to an increase in home-delivered meal service demand in major French cities and probably elsewhere in the world.

A couple of authors have pointed out that elderly people who delegate their food activities (food purchasing, cooking…) have a worse nutritional status than people who take care themselves of their food activities. Maitre et al. (6) observed at home that the proportion of elderly people who were at risk of undernutrition was 8% for autonomous persons, 16% for persons receiving non-food-related help, and 46% for persons receiving food-related help. Crichton et al.'s (7) meta-analysis showed that older people receiving homecare services display the highest malnutrition prevalence of all the community-dwelling elderly sample studied.

In this context, the purpose of the present study was to conduct a systematic litterature review of all studies related to the nutritional issue in home-delivered meal older recipients. This review was expected (i) to shed light on the nutritional status of older people who benefit from home-delivery service to better understand their needs and (ii) to evaluate if home-delivered meal service can be a relevant and effective lever to preserve or improve the nutritional status of older people.

## Methods

Our systematic literature review followed the approach proposed by Arksey and O'Malley (8) as well as the methodology manual published by the Joanna Briggs Institute (9). This methodology summarizes the evidence available on a topic to convey the breadth and depth of that topic. The protocol was drafted using the Preferred Reporting Items for Systematic Reviews and Meta-analysis Protocols (10). A deposit of the protocol was done on HAL: hal-02901422, version 1.

### Research Question

The research question for this review is: “What are the objectives, characteristics and results of existing research conducted on the nutritional issue among older people receiving home-delivered meals (also known as meals-on-wheels)?”

### Eligibility Criteria

The PICOS (Population, Intervention, Comparator, Outcome, Study design) eligibility criteria were as follows (11):

*Population*. Only older adults living at home and receiving home-delivered meal services were eligible. We excluded from the review (1) studies on older people residing at nursing homes or in the hospital and (2) studies that not display specific data and results for home-delivered meal recipients (e.g., studies that display results from a mixed sample including home-delivered meal recipients and recipients of other care services such as home helper or congregate meals).

*Intervention*. Any nutritional intervention was relevant for inclusion (e.g., studies providing additional food items to regular meals-on-wheels, or studies providing dietary guidance). In addition, studies without an intervention (e.g., observational studies) were eligible for inclusion. Any intervention targeting specific disease rehabilitation was excluded (e.g., intervention targeting patients with hypertension, diabetes, cancer).

*Comparators*. Any comparator was relevant for inclusion (e.g., studies comparing home-delivered meal recipients with non-recipients, or studies comparing two types of home-delivered meal services). In addition, studies without a comparator were eligible for inclusion.

*Outcomes*. Two categories of outcomes associated were considered: (1) characterization of the nutritional status (e.g., body mass index—BMI, weight, undernutrition) and (2) characterization of the nutritional intake (e.g., dietary pattern, nutrient intake).

*Study Design*. All types of study design including observational and interventional design as well as all periods of times and duration of follow-up were eligible.

*Others*. No restriction on the date of publication was made. Given the 6-month timeline, only publications written in English were considered for inclusion. Conference abstracts, editorials, narrative review, and non-scientific literature (e.g., articles on websites) were excluded.

### Information Sources and Search Strategy

After repeated attempts and adjustments, a search strategy combining both thesaurus and free-text terms was developed to retrieve articles of interest in the following databases: PubMed, Web of Science (WOS), and EMBASE (Supplementary File 1). Separate title, abstract, and keyword searches were conducted for older people, home-delivered meal service, and nutritional outcomes on June 2020. The results for the three separate search strings were combined to identify relevant titles. Afterward, references from selected articles and systematic reviews were checked manually for further screening in case they have been not identified during the whole search process. After removing the duplicates, titles, abstracts, and full texts were screened by two independent reviewers against the agreed inclusion and exclusion criteria. For each screening level, a training exercise was conducted prior to the starting of the screening process on a random sample of 50 titles (level one screening), 20 abstracts (level two screening), and 10 full-text (level three screening) to ensure high inter-reviewer reliability. Disagreements between reviewers were resolved by consensus or by consulting a third reviewer. The reasons for exclusion were recorded at the full-text stage.

### Charting the Data

A standardized data abstraction form was developed a priori and revised, as needed, after the completion of a training exercise completed on a sample of five articles. All included studies were abstracted by two reviewers, independently, with conflicts resolved by a third reviewer. The data abstraction form included the following items:

- Article identifiers (authors, year of publication)
- Study identifiers (objective, design, country)
- Population (age, gender, sample size, inclusion, and exclusion criteria)
- Intervention (if applicable)
- Comparator (if applicable)
- Outcomes (endpoints, measurement method, main results).

### Quality Assessment

All included studies were assessed for quality by two reviewers, independently, with conflicts resolved by a discussion until consensus was reached. The quality of the articles was assessed by using the quality assessment criteria developed by Kmet et al. (12). The criteria were the following:

1. Is the objective of the study sufficiently described?
2. Is the study design evident and appropriate?
3. Is the method of subject selection described and appropriate?
4. If interventional and random allocation was possible, was it described?
5. If interventional and blinding of investigators was possible, was it reported?
6. If interventional and blinding of subjects was possible, was it reported?
7. Are subject characteristics sufficiently described?
8. Are outcome measures well-defined and robust to measurement?
9. Is the sample size appropriate?
10. Are analytic methods described, justified, and appropriate?
11. Is some estimate of variance reported for main results?
12. Are they controlled for confounding?
13. Are the results reported in sufficient detail?
14. Are the conclusions supported by results?

Each question can be answered with “yes,” “partial,” “no,” and “not applicable.” The associated scoring manual of Kmet et al. (12) was used to calculate the quality score as it is described below:

$$\begin{array}{l} Quality\;score=\frac{2*\left(number\;of{\;}^{\prime }yes^{\prime }\right)+\left(number\;of{\;}^{\prime }partial^{\prime }\right)}{28-2*\left(number\;of{\;}^{\prime }not\;applicable^{\prime }\;\right)} \end{array}$$

In addition, the description quality of HDM service (meal frequency, type of meals, content of meal) was assessed (but not included in the quality score).

### Collating, Summarizing, and Reporting the Results

A descriptive summary of the included studies' characteristics was performed. Tables were created to reflect the overall number of studies included, study designs and settings, publication years, the characteristics of the study populations, the outcomes reported, and the countries where the studies were conducted. In line with systematic literature review guidelines, an assessment of the quality of the included studies was performed (9).

## Results

### General Description of the Systematic Review Article Selection

**Figure 1:**
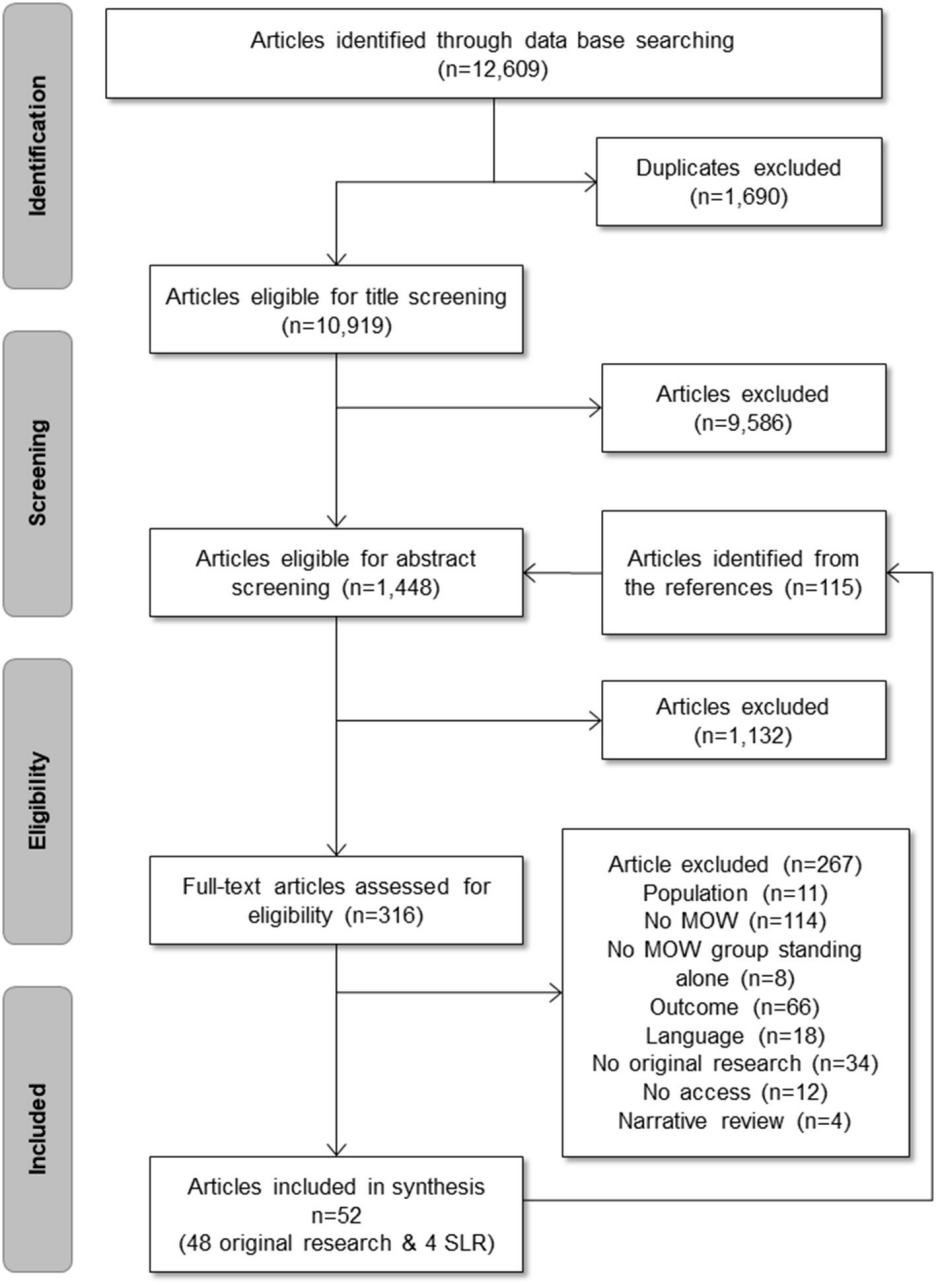
Flowchart.

A total of 10,919 articles were retrieved. After title and abstract screening, 334 records were kept for full-text retrieval and 52 articles were included at full-text review−48 original studies (Figure 1) and four Systematic Literature Reviews (13–16). It should be noted that seven papers including older people with HDM in a broad sample were not included in the synthesis as separated data analyses were not conducted for HDM participants—analyses were done on a broader sample including participants without and with HDM (17–23). Unfortunately, we were not able to find the full text for 12 records despite that we tried to purchase them through the network of university libraries or to contact the authors. Most of these records have old-published years (<1990). The full list of these records can be obtained by contacting the corresponding author of the present paper.

The articles of this selection were published between years 1985 and 2020, most of them being published after 2000 (*n* = 34) (Table 1). More than half of these studies were conducted in the USA (*n* = 32), and two recurrent authors can be identified: Joseph Sharkey and Edward Frongillo. The size of the samples that could be reached varied greatly depending on the studies (ranging from 12 to 4,017 participants). Thirty studies of the selection were cross-sectional, and 18 were longitudinal with follow-up times between 10 days and 2 years (Table 1). In addition, 15 studies implemented a between-subject design while 12 studies implemented a within-subject design; 21 studies were observational (no group nor condition comparison).

**Table 1:** Characteristics of the systematic literature review articles.

**Characteristics**	***n* (%)**
**Year of publication**	
Before 90'	3 (6%)
1990–2000	11 (23%)
2001–2010	18 (37.5%)
2011–2020	16 (33.5%)
**Country**	
Australia	5 (10.5%)
Canada	4 (9%)
Denmark	1 (2%)
Ireland	1 (2%)
Korea	1 (2%)
The Netherlands	2 (4%)
United Kingdom	2 (4%)
United States of America	32 (66.5%)
**Study design**	
Longitudinal study	18 (37, 5%)
* <1 month*	*3 (17%)*
*1–3 months*	*5 (28%)*
*3–8 months*	*6 (33%)*
*≥1 year*	*4 (22%)*
Cross-sectional survey	30 (62.5%)
Between-subject	15
Within-subject	12
Observational	21
**Number of participants**	
>500	11 (23%)
100–500	14 (29%)
50–99	8 (16.5%)
20–49	10 (21%)
<20	5 (10.5%)

After conducting this descriptive summary of the collected studies related to the nutritional issue of home-delivered meal recipients, the three following topics were addressed:

- Characterization of the nutritional risk among older home-delivered meal recipients.
- Comparison of home-delivered meal recipients with non-recipients: does receiving home-delivered meal provide a nutritional benefit compared to when home-delivered meal is not received?
- Improvement of home-delivered meal service: which nutritional interventions are effective to improve the nutritional status of home-delivered meal recipients?

### Methodological Quality

The methodological quality of the included studies was in general good with an average quality score of 0.81 (standard deviation: 0.13) ranging from 0.54 (24) to 1 (25–27) (Appendix 1). Overall, the control of confounding factors and the characterization of participants were poorly rated in the selected studies. This was because most of the studies did not consider all the factors established as possible confounding variables in studies on older adults: age, gender, diseases, drug intake, functional and cognitive status, socioeconomic status, and dental status. The quality variable related to the outcome was also poorly rated, mainly because several studies measured nutrient intake using only one 24-h recalls and not three (2 weekdays and 1 weekend day) as it is generally recommended (28). Finally, the quality variable that was the most poorly rated was the description quality of HDM service: very few studies provided information about the HDM service provided to the participant (How many meals are delivered per week? Which meals are delivered—breakfast, lunch, dinner? What do they contain—starter, main dish, dairy product, dessert.? How are the meals delivered: frozen, cold, hot?).

### Characterization of the Nutritional Risk Among Older Home-Delivered Meal Recipients

Table 2 shows the 22 articles from the systematic literature review, which provide information about the nutritional risk among home-delivered meals recipients.

**Table 2:** Characterization of nutritional risk in HDM applicants and recipients.

**Author(s), year**	**Population**	**Age**	**Nutritional risk**
**Country**		**% women**	
		**% living alone**	
Coulston et al. (29) USA	230 HDM applicants	77.4 ± 7.0 y 68% of women	NSI High nutritional risk 83% Multi-criteria evaluation Risk of undernutrition 74%
Melnik et al. (30) USA	154 eligible older adults for New-York state HDM program	>60 y	NSI High nutritional risk 40%
Borkent et al. (25) The Netherlands	98 newly enrolled HDM recipients	80.4 ± 6.8 61% of women 49% living alone	SNAQ 65+^b^ No risk of undernutrition 80% Moderate risk of undernutrition 10% Severe risk of undernutrition 10%
Kretser et al. (31) USA	203 newly enrolled HDM recipients	60–90 y 72% of women 39% living alone	MNA^b^ Not at risk of undernutrition 5% At risk of undernutrition 69% Undernutrition 26%
Luscombe-Marsh et al. (32) Australia	28 newly enrolled HDM recipients	Age > 60 y 79% of women 57.6% living alone	MNA^b^ Mean: 20.5 ± 2.6 (<24 nutritional risk)
Marceaux (33) USA	40 newly enrolled HDM recipients from Austin	75.3 ± 6.6 y 77% of women >50% living alone	MNA-SF^b^ Not at risk of undernutrition 20% At risk of undernutrition 47.5% Undernutrition 32.5% NSI^b^ Low nutritional risk 0% Moderate nutritional risk 22.5% High nutritional risk 77.5%
Roy and Payette (34) Canada	51 newly enrolled HDM recipients from Sherbrooke	76.4 ± 4.9 y 82% of women 71% living alone	ENS^b^ Low nutritional risk 3,5% Medium nutritional risk 49% High nutritional risk 47.5%
Ullevig et al. (35) USA	49 newly enrolled HDM recipients	77.2 ± 8.2 y 59% of women	NSI^b^ Low nutritional status 2% Moderate nutritional risk 18.5% High nutritional risk 79.5% MNA-SF^b^ Not at risk of undernutrition 21% At risk of undernutrition 37.5% Undernutrition 41.5%
Vailas et al. (36) USA	45 newly enrolled HDM recipients	79.1 ± 7.5 y 73% of women 67% living alone	NSI^b^ Mean: 4.9 ± 2.6 (score ≥ 6 indicates high nutritional risk)
Choi et al. (37) USA	736 HDM clients	70%>60 y 69.7% of women 60% living alone	NSI Mean nutritional risk score 7.6 (3.4) (score ≥ 6 indicates high nutritional risk)
Dewar et al. (38) UK	399 HDM recipients from Hils	83.4 ± 10.9 y 65% of women 74% living alone	MUST^b^ Low risk of undernutrition 56% Medium risk of undernutrition 19% High risk of undernutrition 25%
Fey-Yensan et al. (39) USA	230 HDM recipients from Rhode Island	82.3 y 71% of women 38% living alone	NSI Low nutritional risk 4% Moderate nutritional risk 14% High nutritional risk 82%
Galea et al. (40) Australia	12 HDM recipients from Camden	84.9 ± 10.9 y 83% of women 92% living alone	MNA Not at risk of undernutrition *n*=11; 92% At risk of undernutrition *n*=1; 8%
Herndon (41) USA	245 HDM recipients from Lake County, Indiana	79.3 y 71% of women 56% living alone	NSI Low nutritional risk 28% Moderate or high nutritional risk 72%
Lipschitz et al. (42) USA	33 HDM recipients from Pulaski County	77.3 ± 1.4 y	Multi-criteria evaluation 36% at risk of malnutrition
O'Dwyer et al. (43) Ireland	63 HDM recipients	78.5 ± 10.7 y 59% of women 86% living alone	MNA Not at risk of undernutrition 63.5% At risk of undernutrition 27% Undernutrition 9.5%
Ponza et al. (44) USA	818 HDM recipients	78 y 70% of women 60% living alone	NSI approximation^a^ Low nutritional risk 12% Moderate nutritional risk 40% High nutritional risk 48%
Sharkey (45) USA	429 HDM recipients from North Carolina	78.5 ± 8.0 y 79% of women 58% living alone	NSI approximation^a^ Low/moderate nutritional risk 29% High nutritional risk 70%
Sharkey (46) USA	908 HDM recipients from Texas Lower Rio Grande Valley	60–104 y 62% of women 52% living alone	NSI approximation^a^ Low nutritional risk 3% Moderate nutritional risk 15.5% High nutritional risk 81.5%
Walton et al. (47) Australia	42 HDM recipients from New South Wales	81.9 ± 9.4 y 62% of women 67% living alone	MNA Not at risk of undernutrition 57% At risk of undernutrition 38% Undernutrition 5%
Wright et al. (48) USA	51 HDM recipients from Florida	74.1 y 66% of women	MNA-SF Not a risk of undernutrition 8% At risk of undernutrition 58% Undernutrition 34%
Wunderlich et al. (49) USA	96 HDM recipients from New Jersey	79.0 ± 9.9 y	NSI^b^ Mean: 8.1 (score ≥ 6 indicates high nutritional risk)

*y, years old; HDM, home-delivered meal; MNA, Mini Nutritional Assessment; NSI, Nutrition Screening Initiative; MUST, Malnutrition Universal Screening Tool; SNAQ 65+, Short Nutritional Assessment Questionnaire 65+; ENS, Elderly Nutrition Screening Tool*.

a *NSI approximation: the item on oral trouble was removed from the original NSI checklist, but the authors kept the same thresholds as the ones defined in the NSI*.

b *Data were extracted from baseline data*.

Across the 22 studies, eight different screening methods were used. The Nutrition Screening Initiative checklist (NSI) was the most frequently used [*n* = 12 articles; 24–34, 88]. The NSI has been developed thanks to a collaborative effort between the American Dietetic Association, the American Academy of Family Physicians, and the National Council on the Aging (50, 51). The Mini Nutritional Assessment (MNA) or its short form (MNA-SF) are used in eight articles (31–33, 35, 40, 43, 47, 48). The MNA and MNA-SF are validated tools developed by the International Association of Geriatrics and Gerontology (52). The other tools were the following: the Elderly Nutrition Screening Tool (ENS) (34), the Malnutrition Universal Screening Tool (MUST) (38), and the Short Nutritional Assessment Questionnaire 65+ (SNAQ 65+) (25). Finally, two articles relied on a multi-criterion evaluation to diagnose undernutrition. In Coulston et al. (29), respondents were diagnosed at risk of undernutrition if they met at least one criterion among the following criteria: anthropometric measurements, dietary intakes, and blood sample analysis. In Lipschitz et al. (42), respondents were diagnosed at risk of undernutrition if they met at least one criterion among the following criteria: food intake, ideal body weight, albumin, and Total Iron Binding Capacity.

Nine studies assessed the nutritional status of home-delivered meal applicants or newly enrolled recipients. All these studies but one highlighted the nutritional frailty of this population, with a prevalence for nutritional risk or undernutrition ranging from 79 to 100%. It should be noted that four studies were carried out in the USA, in the context of the Old American Act (OAA) which specifically targets older people with the greatest economic or social need. In the study of Borkent et al. (25) conducted in The Netherlands with the SNAQ 65+ tool, only 20% of newly enrolled HDM were at risk of undernutrition. In parallel, in the study of Sahyoun et al. (53) (*n* = 566), 39% of the older adults receiving HDM after hospital discharge reported a fair or poor appetite, and in the study of Frongillo et al. (54) (*n* = 4,019), 17.5% of the older adults eligible of the HDM program reported not eating for 1 day or more. Luscombe-Marsh et al. (32) as well as Vailas et al. (36) reported respectively average MNA (20.5 ± 2.6) and NSI (4.9 ± 2.6) scores revealing a nutritional risk.

Eleven studies assessed the nutritional status of home-delivered meal recipients. Four studies reported that about 40% of their studied sample were at moderate or high nutritional risk [32.5% in O'Dwyer et al. (43); 36% in Lipschitz et al. (42); 43% in Walton et al. (47); 44% in Dewar et al. (38)]. Six studies (all in the USA) reported a high prevalence for nutritional risk or undernutrition, ranging from 70 to 96% (39, 41, 44–46, 48). In addition, Choi et al. (37) and Wunderlich et al. (49) reported respectively average NSI scores (7.6 and 8.1) corresponding to a high nutritional risk. Only one study reported a very low prevalence for undernutrition (8%), but the studied sample only included 12 participants (40).

In a very interesting study, Melnik et al. (30) randomly selected households in the New York state. If the household included at least one person aged 60 years or older, the authors determine his/her eligibility for HDM (e.g., homebound, lack of family support, unable to shop/to prepare their own meals). Overall, eligibility status was determined for more than 4,500 older adults. Finally, a nutritional assessment was performed in a subset of particularly needy HDM eligible adults (*n* = 146) and in a random subset of non-eligible older adults (*n* = 408). The results showed that 40% of the HDM eligible group were at high nutritional risk (NSI score) against 15% in the non-eligible group.

Five studies reported data on weight loss or underweight. In two large cohort studies conducted in HDM recipients, one from North Carolina, USA (45), *n* = 1,026, and a second one from Texas, USA (46), *n* = 908, respectively 26% and 43% of the respondents reported an unintended weight change of 10 pounds in the last 6 months. Herndon (41) reported that 11% HDM recipient had lost at least 10 pounds (4.5 kg) in the last 6 months. In a study conducted with 244 HDM recipients from New York state, Roe (55) reported that 33 and 11% of the respondents were respectively underweight and overweight. However, O'Dwyer et al. (43) reported that only 3% of HDM recipient were underweight (<18.5 kg/m^2^).

Ten articles explored food intake in HDM recipients—all these studies were conducted in North America but one in Australia (Table 3). Congruently with the nutritional frailty reported in the studies depicted in Table 2, the studies presented in Table 3 highlight that HDM recipients hardly reached the Recommended Daily Allowance (RDA) for energy and in a lesser extent for protein (24, 26, 42, 44, 56–60). For instance, the large survey of Ponza (44) conducted on 818 older adults enrolled in an HDM program funded by OAA showed that energy and protein intakes were below two-thirds of the RDA for 44 and 14% of the sample, respectively. Borkent et al. (25) showed that only 27% of newly enrolled HDM recipients reached an intake of 1.2 g protein/kg body weight/day—the recommended allowance for this population.

**Table 3:** Food intake in HDM recipients.

**Author(s), year**	**Population**	**Age**	**Food intake/appetite**
**Country**		**% women**	
		**% living alone**	
Borkent et al. (25) The Netherlands	98 newly enrolled HDM recipients	80.4 ± 6.8 y 61% of women 49% living alone	71% of respondents did not fulfill the recommendation of 1,2 g of protein / kg of BW / day
Charlton et al. (56) Australia	13 HDM recipients at risk of undernutrition or undernourished	81.3 ± 10.9 y 58% of women 58% living alone	On average, energy and protein intakes are lower than the RDA (baseline data)
Frongillo et al. (57) USA	1,505 HDM recipients from New-York City	Age > 60 y 72.6% of women 71% living alone	HDM participants consumed less fruits, vegetables, and milk than the recommended frequency of consumption
Hoogenboom et al. (58) USA	61 HDM recipients from East Central Indiana	age > 55 y 67% of women	Daily energy intake was lower than the RDA
Krondl et al. (59) Canada	392 HDM recipients from Southern Ontario	82.0 ± 5.4 y 66% of women 74% living alone	For both men and women, energy intake was below the average energy requirements Protein intake was slightly below the RDA in women only
Lipschitz et al. (42) USA	33 HDM recipients from Pulaski County	77.3 ± 1.4 y	35% of the HDM recipients did not meet 80% of the energy and protein RDA (baseline data)
MacLellan (60) Canada	20 HDM recipients from Charlottetown	81.4 ± 6.9 y 55% of women 75% living alone	Energy intake was below the RDA in men only Both men and women met the RDA for protein
Ponza et al. (44) USA	818 HDM recipients	78 y 70% of women 60% living alone	Energy and protein intakes were below two-thirds of the RDA for 44 and 14% of the sample, respectively
Sharkey (26) USA	279 HDM female recipients from North Carolina	79 y 100% of women 58% living alone	Energy intake was below two-thirds of the RDA for 25% of the sample Protein intake was below the RDA for 25% of the sample
Walden et al. (24) USA	20 Random sample of HDM recipients	81.4 ± 8.7 y 75% of women 81% living alone	56% of the participants did not meet the RDA for energy intake; 6% for protein intake

*y, years old; HDM, home-delivered meal; RDA, recommended daily allowance; BW, body weight*.

Interestingly, Foglerlevitt et al. (61) measured the rate of consumption of delivered meals by HDM recipients and observed that meal utilization of the delivered meals was 81% for energy and 83% for protein. Furthermore, Galea et al. (40) observed that home-delivered meals met the nutritional recommended guidelines in terms of energy and protein, but only if all the three components of the meal were ordered (soup, main dish, dessert).

### Comparison of Nutritional Outcomes Between Recipients and Non-recipients of Home-Delivered Meal Service

Eighteen studies compared the nutritional outcomes (food and/or nutrient intakes, body weight, or nutritional status) between a situation where the older participants are receiving home-delivered meals and a situation where participants are not receiving home-delivered meals (Table 4). These studies include one randomized control trial (62), eight parallel-group design studies (3, 4, 32, 34, 63–66), and two cross-sectional studies (67, 68), all of these studies comparing a HDM group vs. a non-HDM group. Four pre–post studies (33, 35, 48, 69) included newly enrolled HDM recipients to be followed up over 2 or 3 months after implementing a home-delivery meal service. Finally, three within-subject design studies (24, 47, 70) compared food intake between 1 day with HDM and 1 day without HDM.

**Table 4:** Comparison of nutritional outcomes between recipients and non-recipients of home-delivered meal service.

**Author(s), year**	**Design Follow-up**	**Population**	**Intervention**	**Comparator**	**Main results**
**Country**					
Buys et al. (62) USA	RCT Cross-sectional	Patients at risk of undernutrition from hospital discharge 77.2 ± 9.6 y	Delivery of 3 meals per day and distribution of booklet with nutritional advices *n* = 11	Distribution of booklet with nutritional advices *n* = 19	Energy intake: higher in the HDM group than in the control group
Arjuna et al. (63) Australia	Parallel group 12 weeks	Older adults at risk of undernutrition 83.1 ± 1.1 y	HDM recipients receiving at least 3 meals/week (~550 kcal; 30 g protein) *n* = 16	Participants eligible for HDM but not receiving it *n* = 11	Nutrient intake, nutritional status, BW: no change in both groups compared to baseline
Denissen et al. (3) The Netherlands	Parallel group 3 months	Older adults, functionally disabled receiving home care age >70 y	Delivery of high-quality meals for 4–7 days/week. Meals were prepared using fresh ingredients according to dietary guidelines *n* = 16	No HDM *n* = 14	Energy and protein intakes: no change in both groups compared to baseline BW: increase in both groups, but higher increase in the HDM group than in the control group
Frongillo and Wolfe (64) USA	Parallel group 1 year	New home-care service recipients 60–100 y	Implementation of HDM *n* = 171	Implementation of home-care services but no HDM *n* = 41	In the HDM group: fruit and vegetable variety increase compared to baseline. Energy and protein intake, BW: no change compared to baseline Dairy and vegetable intake: higher in the HDM group than in the control group at 6 months (but not at 12 months)
Keller (65) Canada	Parallel group 18 months	Home-care service recipients 78.7 ± 8.0 y	HDM recipients *n* = 74	No HDM *n* = 189	Nutritional status (SCREEN): higher in the HDM group than in the control group at follow-up
Lindhart and Nielsen (66) Denmark	Parallel group 12 weeks	Patients at risk of undernutrition from hospital discharge 79.4 ± 8.4 y	Delivery of high energy and high protein meal *n* = 9	No HDM *n* = 16	BW: no change in both groups compared to baseline
Luscombe-Marsh et al. (32) Australia	Parallel group 12–15 months	Home-care service recipients at risk of undernutrition 69–99 y	Implementation of HDM *n* = 28	No implementation of HDM *n* = 80	Self-reported weight loss: no difference between HDM and control group
Park and Son (4) Korea	Parallel group 8 months	Older women living alone in a low-income area near Seoul age>65 y	Undernourished women receiving home-food delivery *n* = 22	Well-nourished participants without home-food delivery *n* = 22	Meat, fish, eggs, soybean products, vegetable and fruit intake: higher in the intervention group than in the control group at follow-up Energy and protein intakes, BW: no change compared to baseline in both groups
Roy and Payette (34) Canada	Parallel group 8 weeks	Applicants for receiving food related home help 76.4 ± 4.9 y	Implementation of HDM *n* = 20	No implementation of HDM *n* = 31	Energy and protein intakes: increase in the HDM group compared to baseline; no change in the control group
Prothro et al. (67) Korea	Between-subject Cross-sectional	Meal-help recipients 78.6 ± 8.4 y	HDM recipients *n* = 51	Congregate meal recipients *n* = 52	BW: lower in the HDM group than in the congregate-meal group
Steele and Bryan (68) USA	Between-subject Cross-sectional	Homebound HDM recipients 79.4 ± 7.0 y	Implementation of HDM (1 meal/day, 5 days/week) *n* = 32	No implementation of HDM *n* = 22	Carbohydrate, thiamin, and iron intakes: lower in the HDM group than in the control group Energy, protein, and fat intakes: no difference between the groups
Marceaux (33) USA	Pre–post 3 months	New HDM recipients *n* = 40 65–96 y	Implementation of HDM service	N/A	Protein intake: increase compared to baseline; energy intake: no change Nutritional status (MNA):77% of the undernourished participants and 31% of the participants at risk of undernutrition improved their nutritional status
O'Leary et al. (69) UK	Pre–post 3 weeks	Community-dwelling older adults *n* = 19 78.3 ± 8.7 y	Delivery of 3 meals a day + snacks available *ad-libitum*	N/A	Nutritional status (MNA): improvement compared to baseline data BW: no change
Ullevig et al. (35) USA	Pre–post 3 months	New HDM recipients *n* = 79 77.2 ± 8.2 y	Implementation of HDM service	N/A	Nutritional status (NSI and MNA-SF): improvement compared to baseline Nutrient intakes: no change
Wright et al. (48) USA	Pre–post 2 months	New HDM recipients *n* = 62 74.1 y	Implementation of HDM service (3 meals/week at minimum)	N/A	Energy and protein intakes: increase compared to baseline Nutritional status (MNA-SF): improvement compared to baseline
An (70) USA	Within-subject Cross-sectional	HDM recipients *n* = 146 73% > 60 y	One day with HDM	One day not without HDM	Protein, fiber, calcium intakes: higher during the HDM day than during the non-HDM day Energy intake: no difference
Walden et al. (24) USA	Within-subject Cross-sectional	Random sample of HDM recipients *n* = 20 81.4 ± 8.7 y	One day with HDM	One day without HDM	Energy, carbohydrates, and fat intakes: higher during the HDM day than during the non-HDM day
Walton et al. (47) Australia	Within-subject Cross-sectional	HDM recipients *n* = 42 81.9 ± 9.4 y	One day with HDM	One day without HDM	Energy intake: higher during the HDM day than during the non-HDM day for women only

*y, years old; RCT, randomized controlled trial; HDM, home-delivered meal; N/A, not applicable. MNA, Mini Nutritional Assessment; NSI, Nutrition Screening Initiative; SCREEN, Risk Evaluation for Eating and Nutrition; BW, body weight*.

Six articles reported an improvement of energy intake with HDM (24, 34, 47, 48, 62, 64) while six observed no difference between a HDM vs. non-HDM situation (3, 33, 35, 63, 68, 70). Six articles reported an improvement of protein intake with HDM (33, 34, 48, 64, 70), and 1 observed no differences between a HDM vs. non-HDM situation (47). In parallel, Frongillo and Wolfe (64) and Park and Son (4) reported higher meat consumption in HDM recipients than in non-HDM participants. Interestingly, the three within-subject articles showed higher energy and/or protein intakes when participants received a home-delivered meal than when they cooked their own meal (24, 47, 70).

None of the studies observed an improvement of body weight with HDM (4, 32, 64, 66, 67, 69) except for Denissen et al.'s (3). The latter observed a higher body weight increase in participants who have received high-quality HDM for 3 months (nutritious and appealing meals prepared with fresh ingredients) compared to participants who did not receive HDM meanwhile. Interestingly, Prothro and Rosenbloom (67) observed lower body weight in HDM recipients compared to older adults taking part in congregate meals.

Finally, all the studies that have looked at the nutritional status observed a decrease of the nutritional risk with HDM (33, 35, 48, 65, 69). For instance, Marceaux (33) observed in newly enrolled HDM recipients that 31% of the participants at risk of undernutrition and 77% of the undernourished participants improved their nutritional status after receiving HDM for 3 months.

### Impact of an Improved Home-Delivered Meal Service on Nutritional Outcomes Compared to a Regular Service

Nine studies assessed the impact of an “improved” HDM service on nutritional outcomes compared to a regular service (Table 5). Improvement consisted in providing energy and/or protein enriched meals and/or snacks [six studies: 21, 44, 45, 49, 56, 64], in providing additional meals and/or snack (e.g., breakfast) to the regular offer (31, 71), or in providing dietetic counseling (49).

**Table 5:** Impact of an improved HDM service on nutritional outcome compared to a regular service.

**Author(s), year**	**Design**	**Population**	**Intervention**	**Comparator**	**Main results**
**Country**	**Follow-up**				
Arjuna et al. (63) Australia	Parallel group 12 weeks	HDM recipients at risk of undernutrition 83.1 ± 1.1 y	Enriched HDM (~1,100 kcal; 60 g of protein) for at least 3 days/week *n* = 14	Standard HDM (~550 kcal; 30 g of protein) for at least 3 days/week *n* = 16	Energy and protein intakes, nutritional status (MNA): increase with enriched HDM compared to baseline; no change with standard HDM BW: no change whatever the group compared to baseline
Borkent et al. (25) The Netherlands	RCT 4 weeks	New HDM recipients 80.4 ± 6.8 y	Daily delivery of a protein-enriched hot meal (~30 g of protein) and a dairy product *n* = 49	Daly delivery of a regular hot meal (~21 g of protein) and a drink *n* = 49	Protein intake: no change with the enriched HDM while it decreases with standard HDM compared to baseline Energy intake: no change in both groups compared to baseline
Charlton et al. (56) Australia	Pre–post 4 weeks	HDM recipients at risk of undernutrition or undernourished *n* = 13 81.3 ± 10.9 y	Delivery of sweet and savory enriched snacks five times/week in addition to regular HDM	N/A	Protein intake: tended to increase compared to baseline Energy intake: no change Nutritional status (MNA): improvement BW: increase
Dewar et al. (38) UK	Pre-post 6 months	HDM recipients *n* = 399 83.4 ± 10.9 y	Delivery of additional snacks (soups, cream tea, scones: 150–500 kcal)	N/A	Nutritional status (NSI): 90% of the population maintained or improved their NSI score compared to baseline
Gollub et al. (71) USA	Within-subject Cross-sectional	HDM recipients since at least 6 months, at risk of undernutrition 60–100 y	Delivery of a breakfast in addition to 5 lunches/week *n* = 167	Delivery of 5 lunches/week *n* = 214	Energy and protein intakes: higher when breakfast was provided than in the control condition
Kretser et al. (31) USA	RCT 6 months	New HDM recipients 60–90 y	Daily delivery of three meals (breakfast, lunch, dinner) and two snacks 7 days/week *n* = 61	Delivery of a hot meal 5 days/week *n* = 56	Nutritional status (MNA): improvement in both groups compared to baseline BW: increase with the improved HDM offer; no change in the control group compared to baseline
Lipschitz et al. (42) USA	Pre–post 16 weeks	HDM recipients, at risk of undernutrition *n* = 12 77.3 ± 1.4 y	Delivery of a polymeric dietary supplement in addition to HDM	N/A	Energy and protein intake: increase compared to baseline Nutritional status (serum albumin): improvement BW: no change
Silver et al. (72) USA	Within-subject 1 week	HDM recipients *n* = 45 84.4 ± 1.0 y	Delivery of energy and protein enriched lunches (energy density twice the regular version and 10 g more protein per serving)	Delivery of regular lunches (~1/3 of the RDA for energy)	Energy and protein intakes: higher with enriched lunches than with regular lunches
Wunderlich et al. (49) USA	Pre–post 2 years	HDM recipients *n* = 96 79.0 ± 9.9 y	Nutrition education and dietetic counseling	N/A	Nutritional status (NSI): improvement compared to baseline data

*y, years old; RCT, randomized controlled trial; HDM, home-delivered meal; N/A, not applicable. MNA, Mini Nutritional Assessment; NSI, Nutrition Screening Initiative; BW, body weight; RDA, recommended daily allowance*.

Providing enriched home-delivered meals led to a stabilization (25) or an increase of energy and/or protein intake as well as to an improvement of the nutritional status (38, 42, 56, 63). For instance, in Arjuna et al. (63), HDM recipients at risk of undernutrition in the improved condition were delivered with meals containing twice as much energy and protein as the regular version. After 12 weeks, results showed an increase of energy and protein intakes as well as a decrease of the nutritional risk (MNA) with the enriched HDM offer while no change was observed with the regular HDM offer. Borkent et al. (25) observed that protein intake remained stable when newly enrolled HDM recipients were provided with enriched hot meals while it decreased with the provision of a regular hot meal. However, provided enriched meals do not guarantee that people will meet the recommended nutritional allowance. In Borkent et al. (25), about two-thirds of the enriched HDM group did not reach the threshold of 1.2 g protein/kg of body weight/day (they were about 90% in the regular HDM group). A similar result was observed for energy intake in Charlton et al. (56).

Providing three meals and two snacks seven days per week led to an increase of body weight compared to HDM recipients who received a hot meal 5 days per week (31). Providing breakfasts in addition to lunches led to an increase of energy and protein intake compared to HDM recipients who received only lunches (71). Finally, providing dietetic counseling led to an improvement of the nutritional status (NSI) in HDM recipients after 2 years of follow-up (49).

## Discussion

Literature inquiry led to the identification of four systematic literature reviews close to the scope of the present review. The systematic review of Campbell et al. (13) targeted all studies related to home-delivered meals and included 80 articles. This literature review did not focus on older adults (the review included studies on people aged 45+ years). Included articles were sorted according to their experimental design, each design including various outcomes (e.g., satisfaction, food insecurity, nutritional outcomes, health outcomes, care expenditures). Campbell et al. (13) provided few conclusions about the nutritional issue in home-delivered meal recipients. Rather, they provided a detailed overview of the different types of studies that have been conducted on home-delivered meal services and recipients. Two systematic literature reviews addressed a topic close to our second question (comparison of home-delivered meal recipients with non-recipients). However, the one of Zhu and An (16) was restricted to studies conducted in the USA, in relationship with the Older Americans Act. This review concluded that the US home-delivered meal programs improve diet and increase nutrient intakes among recipients. The recent review of Walton et al. (15) assessed the impact of receiving meal services on nutritional intake compared to when no meal services are received in older adults living at home. In this review, meal services were not only restricted to home-delivered meals but also included congregate meals. The results highlighted a positive impact of home-delivered meals on energy and protein intake in older adults. However, this review did not explore the impact of meal services on the nutritional status and the undernutrition risk among the older population. Finally, the systematic literature review of IJmker-Hemink et al. (14) explored the effectiveness of various interventions to improve nutrition and satisfaction outcomes in adults receiving home-delivered meals. This review led to the identification of 12 studies assessing the impact of an improved home-delivered meal service on nutritional outcomes (14 assessed satisfaction outcomes). However, in their review, the authors did not separate the studies assessing the impact of providing HDM service vs. no HDM service (as we did on Table 4) from assessing the impact of an improved HDM service (e.g., with dietetic counseling, enriched meal) vs. providing a standard HDM service (as we did on Table 5).

The present review aimed at compiling all the studies related to the nutritional issue in home-delivered meal older recipients. Whether older people are newly enrolled in an HDM service or are already beneficiary of an HDM service, the prevalence of the nutritional risk is high or even very high. Over the 19 studies included in our review, 15 studies displayed a prevalence higher than 35%—higher than 70% in 10 studies. In parallel, nine studies highlighted that HDM recipients hardly reached the Recommended Daily Allowance for energy and to a lesser extent for protein. In fact, this service is rarely a comfort service, but it generally counterweights the difficulties encountered by older people in feeding themselves (73). Several authors reported that older beneficiaries of HDM service have less social contact and are less mobile compared to the general population (1, 53, 74, 75). In numerous countries (and in particular in the USA where the majority of the studies were conducted), the HDM service often targets older people with a low socioeconomic status, with high levels of food insecurity (76, 77). All these factors—loneliness, functional disabilities, poor financial resources—are known to be risk factors for undernutrition (37, 78–80).

Results of the present literature review highlight that providing older people with HDM service improves their nutritional status, leading to a decrease in the undernutrition prevalence. Most of the studies also show that providing home-delivered meals leads to a higher energy intake (seven studies over 12) and protein intake (five studies over six). None reported a decline in dietary intake. These results are in line with previous literature reviews' findings. Zhu and An (16) concluded that the US home-delivered meal programs improve diet and increase nutrient intakes among recipients. Similarly, Walton et al. (15) highlighted a positive impact of home-delivered meals on energy and protein intake in older adults. In addition, several studies showed that improving the HDM service by providing dietetic counseling to the beneficiaries, or providing additional snacks/meals, or providing calorie and/or protein enriched dishes improved the nutritional status and/or dietary intakes compared to a regular HDM service (31, 38, 42, 49, 56, 63, 71). However, providing an improved home-delivered meal service was not always enough to allow all the older recipients meeting the recommended nutritional allowance (25, 56). For instance, in Borkent et al. (25), about two-thirds of the enriched home-delivered meal group did not reach the threshold of 1.2 g protein/kg of body weight/day (81). This raises the importance of further developing new enrichment solutions to fill this gap.

It should also be noted that all these studies aimed at improving the nutritional content of home-delivered meals. However, further research should also consider improving the sensory quality of the home-delivered foods. In line with the studies carried out by Sulmont-Rossé et al. (82), it would be interesting to optimize the sensory quality of the meals by recruiting the tasting panel among HDM recipients. Improving meals according to older people's feedback led to an increase of food intake in nursing home (83). In addition, improvements could also target the quality of the service such as providing choice and providing social support during the meal. In addition, it can be hypothesized that the combination of several levers is necessary to have significant improvements in food intake rather than implementing only one lever in the fields. In fact, such multidimensional interventions were implemented in nursing homes and proved to be efficient (83–87). For instance, Kremer et al. (86) combined improvement at the product level (e.g., attractive visual presentation), at the people level (e.g., promoting choice to give the older residents more control), and at the situation level (e.g., promoting an attractive social environment) to improve the meals in nursing homes. Such improvements led to an increase in food intake (and meal enjoyment) compared to a regular nursing home setting.

## Limitations and Strengths of the Present SLR

The main strength of this work is that it is a solid literature search, with a complete overview of nutritional issues in home-delivery meal older recipients. A limitation of this review is that we were not able to find the full text for 12 records, mainly because of old published years (<1990) and despite the fact that several channels were used to find them (research on the web, order from university libraries, contact of the authors… none of these articles were available online). From a methodological point of view, an important limitation highlighted by the quality evaluation is the lack of information provided by the authors on the home-delivered meal service. Most of the studies did not indicate which meals are delivered to their participants (e.g., breakfast, lunch, dinner), how often in a week they are delivered, their content (e.g., starter, main dish, dessert; nutrient content), or their presentation (e.g., portion or batch, frozen or cold or hot). However, these factors may impact the outcome variables (nutritional status, food intake) measured in the studies and possibly explain divergences observed between the results of different studies. For instance, Galea et al. (40) highlighted that home-delivered meals meet the recommended guidelines in terms of protein and energy intake, but only if the three components of the meal are ordered (main dish, soup, dessert).

Another limitation to compare the studies is the large variety of tools used to assess the nutritional risk, as it was already highlighted by the systematic review on undernutrition diagnosis tools (88). In the present review, eight different screening tools can be listed from the included studies. The most used is the Nutritional Screening Initiative (NSI) checklist, but it is only used in the US studies. Interestingly, this tool was developed in the USA based on consensus between health and social care professionals (89). Initially, this tool aimed at raising caregivers' awareness about the importance of the nutritional risk among the aged population. However, this tool is also used to screen for older adults at risk of undernutrition (50), as it is the case in the studies included in our review.

In parallel, it should be noted that most of the studies were carried out in the USA (32 over 48) which prevents the generalization of the present conclusions to other countries. Finally, it is interesting to note that very few changes in body weight were observed in longitudinal or cohort studies. Among the review selection, only one article reported a higher weight of the home-delivered meal participants in comparison to participants without home-delivered meal (3) and only two articles showed that an improved home-delivered meals service led to an increase of body weight over the time (31, 56). In eight studies, no changes in the body weight were observed, even in the long follow-up [e.g., 12 weeks in Arjuna et al. (63); 16 weeks in Lipschitz et al. (42)]. This questions the relevance of weight change as an outcome variable in such study and/or the way this outcome variable is measured [for instance, in Arjuna et al. (63), participants were weighted while they wore “light clothing,” which may have induced variability].

## Conclusion

The main findings of this review can be summarized in the following points: (1) home-delivery meal older recipients are at high risk of undernutrition; (2) providing home-delivery meals may improve the nutritional status and nutrient intake; (3) this improvement is even higher when the home-delivery meal service is improved, for instance by providing dietetic counseling and adding supplementary snacks/meals or calorie- and/or protein-enriched food. However, even an improved service does not allow all the older recipients meeting their recommended nutritional allowance. The implication of these results for the structures providing home-delivered meals services is to develop innovative strategies to allow their recipients fulfilling their nutritional needs. These strategies should probably take into account both sensory issues (e.g., improving the appealing and palatability of meals), nutritional issues (e.g., providing enriched food), and the psycho-social issue (e.g., providing choice to give the older recipients more control). Finally, the implication of this review for the research is to carry out more randomized control trials and to provide a more precise description of the home-delivered service provided to the recipients to better take into account the meal frequency and content in the results.

## Data Availability Statement

The original contributions presented in the study are included in the article/Supplementary Material, further inquiries can be directed to the corresponding author/s.

## Author Contributions

SF: conceptualization, research, resource provision, data collection, writing original version, and visualization. PT: review and correction. JR, CM, and OM: research and data collection. VV: conceptualization, validation, review, and correction. CS-R: conceptualization, validation, review and correction, and visualization. All authors contributed to the article and approved the submitted version.

## Conflict of Interest

SF and PT were employed by company Saveurs et Vie. The remaining authors declare that the research was conducted in the absence of any commercial or financial relationships that could be construed as a potential conflict of interest.
