# Supplementary material for: The Nutritional Issue of Older People Receiving Home-Delivered Meals: A Systematic Review
Source: Front Nutr. 2021 Mar 4;8:629580. doi: 10.3389/fnut.2021.629580 (PMC7982843; doi:10.3389/fnut.2021.629580)
Supplement: Supplementary file 1 [file Data_Sheet_1.docx]

### Supplementary File 1. Search strategy (For PubMed and EMBASE, thesaurus terms are in bold).

**Older people**

**‘Aged’** OR ‘Elderly’ OR ‘elderlies’ OR ‘older’ OR ‘elder’ OR ‘third age’ OR ‘senior’ OR ‘aging’ OR ‘ageing’ OR ‘old person’ OR ‘old people’ (PubMed, WOS)

**‘Aged’** OR ‘aged patient’ OR ‘aged people’ or ‘aged person’ OR ‘aged subject’ OR ‘elderly’ OR ‘elderly patient’ OR ‘elderly people’ OR ‘elderly person’ OR ‘elderly subject’ OR ‘senior citizen’ OR ‘senium’ (EMBASE)

AND

**Home delivered meal**

‘Meal on the wheel’ OR ‘meal on wheel’ OR ‘meals on wheels’ OR ‘food portage’ OR ‘food delivery’ OR ‘food deliveries’ OR ‘delivered food’ OR ‘meal delivery’ OR ‘meal deliveries’ OR ‘delivered meal’ OR **‘food service’** OR **‘home care service’** OR ‘home meal services’ (Pubmed, WOS)

**'Home delivered meal'** OR 'home delivered meal' OR 'home delivery meal' OR 'meal on wheels' OR 'meals on wheels' OR **'home care'** OR 'domiciliary care' OR 'home care' OR 'home care agencies' OR 'home care program' OR 'home care programme' OR 'home care service' OR 'home care services' OR 'home health care' OR 'home health nursing' OR 'home help' OR 'home nursing' OR 'home service' OR 'homecare' OR 'homemaker services' OR 'home care services, hospital-based' (EMBASE)

AND

**Nutritional outcomes**

**‘Nutritional status’** OR **'Body weight'** OR ‘weight’ OR **‘Body Mass Index’** OR ‘BMI’ OR ‘muscle mass’ OR ‘muscular mass’ OR ‘undernutrition’ OR ‘undernourished’ OR **‘malnutrition’** OR ‘malnourished’ OR ‘denutrition’ OR **‘appetite’** OR ‘diet pattern’ OR ‘dietary pattern’ OR ‘food intake’ OR ‘nutritional intake’ OR ‘protein intake’ OR ‘**energy intake’** OR ‘calories intake’ (PubMed, WOS)

**'Body weight'** OR 'body weight' OR 'total body weight' OR 'weight, body' OR **'body mass'** OR 'bmi (body mass index)' OR 'body mass' OR 'body mass index' OR **'muscle mass'** OR 'muscle mass' OR 'muscle volume' OR 'muscle weight' OR 'weight, muscle' OR **'malnutrition'** OR 'deficient nutrition' OR 'malnourishment' OR 'malnutrition' OR 'underfeeding' OR 'undernourishment' OR 'undernutrition' OR **'dietary pattern'** OR 'dietary pattern' OR 'diet pattern' OR **'food intake'** OR 'food consumption' OR 'food ingestion' OR 'food intake' OR 'meal ingestion' OR 'feed intake' OR **'caloric intake'** OR 'caloric intake' OR 'calorie intake' OR 'calory intake' OR 'energy intake' OR 'intake, caloric' OR 'dietary energy' OR **'protein intake'** OR 'dietary protein' OR 'dietary proteins' OR 'food protein' OR 'intake, protein' OR 'protein consumption' OR 'protein intake' OR 'protein nutrition' OR 'protein feeding' OR 'diet protein' OR 'diet, protein' (EMBASE)
